# Chronic Effects of Static Stretching Exercises on Muscle Strength and Power in Healthy Individuals Across the Lifespan: A Systematic Review with Multi-level Meta-analysis

**DOI:** 10.1007/s40279-022-01806-9

**Published:** 2023-01-31

**Authors:** Fabian Arntz, Adrian Markov, David G. Behm, Martin Behrens, Yassine Negra, Masatoshi Nakamura, Jason Moran, Helmi Chaabene

**Affiliations:** 1 Division of Social and Preventive Medicine, University of Potsdam, Am Mühlenberg 9, 14476 Potsdam, Germany; 2 Division of Training and Movement Sciences, Research Focus Cognition Sciences, University of Potsdam, Am Neuen Palais 10, Building 12, 14469 Potsdam, Germany; 3 School of Human Kinetics and Recreation, Memorial University of Newfoundland, St. John’s, NL A1C 5S7 Canada; 4 Department of Sport Science, Institute III, Otto-Von-Guericke University Magdeburg, Magdeburg, Germany; 5 Department of Orthopedics, Rostock University Medical Center, Rostock, Germany; 6 Research Unit (UR17JS01) “Sports Performance, Health and Society”, Higher Institute of Sport and Physical Education of Ksar Saïd, University of Manouba, Manouba, Tunisia; 7 Faculty of Rehabilitation Sciences, Nishi Kyushu University, 4490-9 Ozaki, Kanzaki, Saga Japan; 8 School of Sport, Rehabilitation and Exercise Sciences, University of Essex, Colchester, Essex UK; 9 High Institute of Sports and Physical Education of Kef, University of Jendouba, 7100 Kef, Tunisia

## Abstract

**Background:**

The current literature on the chronic effects of static stretching (SS) exercises on muscle strength and power is unclear and controversial.

**Objective:**

We aimed to examine the chronic effects of SS exercises on muscle strength and power as well as flexibility in healthy individuals across the lifespan.

**Design:**

Systematic review with meta-analysis of (randomized) controlled trials.

**Data Sources:**

A systematic literature search was conducted in the databases PubMed, Web of Science, Cochrane Library, and SPORTDiscus up to May 2022.

**Eligibility Criteria for Selecting Studies:**

We included studies that investigated the chronic effects of SS exercises on at least one muscle strength and power outcome compared to an active/passive control group or the contralateral leg (i.e., using between- or within-study designs, respectively) in healthy individuals, irrespective of age, sex, and training status.

**Results:**

The main findings of 41 studies indicated trivial-to-small positive effects of chronic SS exercises on muscle strength (standardized mean difference [SMD] = 0.21, [95% confidence interval 0.10–0.32], *p* = 0.001) and power (SMD = 0.19, 95% confidence interval 0.12–0.26], *p* < 0.001). For flexibility, moderate-to-large increases were observed (SMD = 0.96, [95% confidence interval 0.70–1.22], *p* < 0.001). Subgroup analyses, taking the participants’ training status into account, revealed a larger muscle strength improvement for sedentary (SMD = 0.58, *p* < 0.001) compared with recreationally active participants (SMD = 0.16, *p* = 0.029). Additionally, larger flexibility gains were observed following passive (SMD = 0.97, *p* < 0.001) compared with active SS exercises (SMD = 0.59, *p* = 0.001). The chronic effects of SS on muscle strength were moderated by the proportion of female individuals in the sample (*β* = 0.004, *p* = 0.042), with higher proportions experiencing larger gains. Other moderating variables included mean age (*β* = 0.011, *p* < 0.001), with older individuals showing larger muscle strength gains, and the number of repetitions per stretching exercise and session (*β* = 0.023, *p* = 0.004 and *β* = 0.013, *p* = 0.008, respectively), with more repetitions associated with larger muscle strength improvements. Muscle power was also moderated by mean age (*β* = 0.006, *p* = 0.007) with larger gains in older individuals. The meta-regression analysis indicated larger flexibility gains with more repetitions per session (*β* = 0.094, *p* = 0.016), more time under stretching per session (*β* = 0.090, *p* = 0.026), and more total time under stretching (*β* = 0.078, *p* = 0.034).

**Conclusions:**

The main findings indicated that chronic SS exercises have the potential to improve muscle strength and power. Such improvements appear to benefit sedentary more than recreationally active participants. Likewise, chronic SS exercises result in a marked enhancement in flexibility with larger effects of passive, as compared with active, SS. The results of the meta-regression analysis for muscle strength indicated larger benefits of chronic SS exercises in samples with higher proportions of female, older participants, and a higher number of repetitions per stretching exercise and session. For muscle power, results suggested larger gains for older participants. Regarding flexibility, findings indicated larger benefits following a higher number of repetitions per exercise and a longer time under stretching per session as well as a longer total time under stretching.

## Key Points

**Table:** 

Chronic static stretching exercises have the potential to improve muscle strength and power.
The chronic effects of static stretching exercises on muscle strength depend on the training status with sedentary participants demonstrating larger gains in muscle strength compared with recreationally active participants, with an unclear effect observed in trained participants.
Chronic static stretching exercises seem to induce larger gains in muscle strength in samples with larger proportions of female individuals and promote higher gains in muscle strength and power in older participants.
More repetitions per stretching exercise, and session, seem to induce larger gains in muscle strength.
Flexibility seems to benefit more from passive compared with active static stretching training. Additionally, the meta-regression analysis indicated larger flexibility gains with increased repetitions per session, more time under stretching per session, and more total time under stretching.

## Introduction

Static stretching (SS) is widely used in athletic, fitness, and clinical settings. It consists of a controlled continuous movement to the end range of motion (ROM) of a single joint or multiple joints where the muscle(s) remains in a lengthened position for a specific period of time. Static stretching can be conducted by either contracting the agonist muscles (i.e., active static) or by using external forces such as gravity, the help of a partner, or stretching aids such as elastic bands (i.e., passive static) [1]. Generally, the main intended aims of SS are to increase ROM [2, 3], mitigate injury incidence [1, 4], and improve athletic performance [5–7].

The acute effects of SS on muscle strength and power have received much attention over the last two decades. Ample evidence indicates that single-mode prolonged durations of SS (i.e., > 60 s per muscle group) result in significant and practically relevant acute impairments in muscle strength and power, while single-mode shorter SS durations (i.e., ≤ 60 s per muscle group) only induce trivial impairments on these measures [1, 8]. In addition to this, the few ecologically valid SS studies have indicated that performing short durations (i.e., ≤ 60 s per muscle group) of SS as part of a comprehensive warm-up practice produced no negative or even small positive effects on muscle strength and power [9–11].

While the acute effects of SS exercises on muscle strength and power are generally accepted [1, 8, 12, 13], the chronic effects are, as yet, unclear and controversial. In fact, there are studies showing improvements [7, 14, 15], no effects [16–18], or even negative effects [19, 20] of chronic SS exercises on measures of muscle strength and power. For example, Kokkonen et al. [7] reported that 40 min of SS, three times weekly, for 10 weeks increased lower limb ROM, muscle strength, power, and endurance in untrained and recreationally active young adults aged 22 years. In contrast to this, in healthy male participants aged 18 years, who undertook two daily sessions of SS training over 3 weeks, no effect on maximum voluntary contraction force and rate of force development of the plantar flexors was found [18]. Moreover, there is evidence that SS performed three times a week with a total of ten sessions resulted in a decrease in maximal voluntary eccentric torque of the hamstrings and functional performance (i.e., triple hop test) in healthy male participants aged 23 years [19].

Two previous narrative reviews have attempted to clarify the chronic effects of different types of stretching, including SS, on muscle strength and power [21, 22]. However, both studies appeared to provide insufficient information, resulting in inconclusive findings. To the authors’ knowledge, there is only one systematic review of the literature on the chronic effects of various stretching types on joint ROM and measures of muscle strength and power in healthy young adults [23]. Among the 29 studies included in that analysis, only around half of them showed increased muscle strength/power after stretching training with the remaining studies, indicating no effect and thus substantiating the uncertainty of the two previous narrative reviews [21, 22].

To date, there is no systematic review with meta-analysis addressing the chronic effects of SS exercises on measures of muscle strength and power in healthy individuals, pointing to a void in the current literature. Therefore, it is warranted to conduct a systematic review with meta-analysis on the chronic effects of SS exercises on measures of muscle strength and power. Considering the above-mentioned gaps in the current literature, the primary aim of this systematic review with multi-level meta-analysis was to investigate the chronic effects of SS exercises on measures of muscle strength and power in healthy individuals. While we admit that the chronic effect of SS exercises on flexibility is well established, the moderating effects of key variables such as the type of SS (passive vs active), the intensity (below vs at the point of discomfort vs above the point of discomfort), and the time under SS are yet to be identified. Accordingly, as a secondary aim, we sought to examine the chronic effect of SS exercises on flexibility. Moreover, we were interested in identifying the main SS variables to help develop training prescriptions.

## Methods

This systematic review with meta-analysis was prospectively registered in PROSPERO under the registration number (CRD42022312581) and conducted per the latest Preferred Reporting Items for Systematic Review and Meta-analyses (PRISMA) statements [24].

### Search Strategy

The literature search was conducted independently and separately by two of the authors (FA and AM) in PubMed, SPORTDiscus, Web of Science, and Cochrane Library databases up to May 2022. The search was performed using a Boolean search strategy (operators “AND” and “OR”) and a combination of the following keywords: (“Range of Motion” OR “Joint Range of Motion” OR “Joint Flexibility” OR “Passive Range of Motion” OR “Muscle Stretching Exercises” OR “Active Stretching” OR “Passive Stretching” OR “Static Stretching” OR “Dynamic Stretching” OR “Ballistic Stretching” OR “Isometric Stretching” OR “Proprioceptive Neuromuscular Facilitation” OR “PNF Stretching Exercise”) AND (“Muscle Power” OR “Explosive Strength” OR Power OR “Muscle Strength” OR Strength) AND (“Adolescent” OR “Child” OR “Adult” OR “Young Adult” OR “Older Adults” OR aged OR seniors OR elderly) AND (“controlled trial” OR “randomised controlled trial”). These keywords were determined through a literature review, expert opinion, and controlled vocabulary (e.g., Medical Subject Headings [MeSH]). Of note, we have used keywords related to other stretching modalities in our search strategy to ensure that studies where the primary focus was on those stretching modalities but also included a SS and a control group are covered. All included studies, as well as corresponding meta-analyses, were searched for additional eligible publications in “snowball” searches [25]. Only peer-reviewed publications written in English were considered for inclusion.

### Inclusion and Exclusion Criteria

The inclusion criteria for eligible studies were formulated following the PICOS (Population, Intervention, Comparison, Outcome, Study Design) approach [26]. The following criteria were defined: (1) population: healthy participants, without any restrictions on age, sex, or training status [27], (2) intervention: SS training with a minimum duration of  two weeks [2, 28] (3) comparison: passive control group/contralateral leg, (4) outcome: at least one measure of muscle strength (i.e., tests assessing maximum voluntary contraction torque/force) or muscle power (i.e., tests assessing rapid force production within a short time frame such as countermovement jump height), and (5) study design: (randomized) controlled trials with baseline and follow-up measures (within or between subjects). We excluded studies involving subjects with health issues (e.g., chronic low back pain, injuries), not including an active/passive control group or contralateral leg as comparator, and/or lacking baseline or follow-up data.

### Data Extraction

The data were extracted by FA using a standardized template created with Microsoft Excel. The extracted data were cross-verified by AM. In case of any disagreement regarding extracted information or study eligibility, HC was consulted for clarification.

Of note, all reported measures for muscle strength and power as well as flexibility for all time points above two weeks were included. Thus, if a study reported multiple measures for muscle strength and power, they were all included. Further, if a study reported measures for muscle strength and power during and after the intervention period, they were also included. If data were not reported in a way that allowed the calculation of effect sizes (i.e., mean ± standard deviation, raw data), the respective authors were contacted. In cases where authors did not respond, WebPlotDigitizer (v4.5; Ankit Rohatgi, Melrose, MA, USA; https://apps.automeris.io/wpd/) was used to extract relevant data in studies that reported measures of interest graphically [29].

From all included studies, the following information was extracted: (a) lead author and year of publication; (b) comparator (i.e., within/between subjects); (c) type of SS (i.e., active/passive/mixed), (d) participants’ training status [27]; (e) percentage of female individuals in the sample; (f) mean age of participants; (g) mean time under SS per exercise; (h) number of repetitions per SS exercise; (i) number of SS exercises per session^1^; (j) weekly session frequency; (k) intervention period; and (l) SS intensity (i.e., below the point of discomfort [no pain]; at the point of discomfort [moderate pain]; above the point of discomfort [severe pain]). Based on that, we calculated (m) the number of repetitions per session,^2^ (n) time under SS per session, (o) weekly time under SS, and (p) total time under SS. In addition to extracting measures for muscle strength and power, data regarding flexibility (e.g., ROM) were retrieved as a secondary outcome from all included studies.

### Methodological Quality of the Included Studies

The Physiotherapy Evidence Database (PEDro) scale was used to evaluate the methodological quality of the eligible studies. The PEDro scale’s reliability and validity have been previously established [30, 31] as well as its agreement with other assessment tools such as the Cochrane risk of bias tool [32]. Assessment of the methodological quality of the included studies was conducted separately by two authors (FA and AM) and any disagreement was solved by contacting a third author (HC). As blinding of participants, therapists, and assessors is to some extent contrary to the nature of the investigated interventions, and thus, is rarely implemented and reported, items 5–7 were removed as in recently published systematic reviews [33, 34]. Further, item 3 (i.e., “allocation was concealed”) was removed for studies implementing within-subject intervention designs, as each participant received the intervention on one leg while the contralateral leg served as the control. Accordingly, methodological quality was judged regarding the percent of satisfied items (PEDro percent), to allow comparability of studies. This value was further analyzed using meta-regression statistics to assess possible moderating effects of study quality [35]. Additionally, overall funnel plots [36], as well as graphical display of study heterogeneity plots [37] were used to visualize publication bias and heterogeneity. To account for potential differences between study designs, a subgroup analysis of within- versus between-subject designs was conducted for each outcome (i.e., muscle strength, muscle power, and flexibility).

### Synthesis and Analyses

Meta-analyses were performed using the ‘metafor’ [38] and ‘tidyverse’ [39] packages in R (v 4.1.2; R Core Team, R Foundation for Statistical Computing, Vienna, Austria; https://www.r-project.org/). All analyses are available in the supplementary documentation (https://osf.io/gu9w6/). To calculate standardized mean differences, the standardized mean change was calculated using the baseline and follow-up means and standard deviations of the SS training and control groups/contralateral leg. Further, the corresponding variance was calculated as the sum of variances from both groups/contralateral leg [40]. The magnitude of the effect size was interpreted in accordance with Cohen’s thresholds [41]: trivial (< 0.2), small (0.2 to < 0.5), moderate (0.5 to < 0.8), and large (≥ 0.8).

Multilevel mixed-effects meta-analyses were used to calculate the effect size with study and intra-study groups as random effects to examine the chronic effect of SS exercises on muscle strength, muscle power, and flexibility. Further, cluster robust models were calculated using 95% confidence intervals (CIs) and weighted by inverse sampling variance to account for the within- and between-study variance. Restricted maximal likelihood estimation was applied in all models. In addition to the cluster robust models’ point estimates and 95% CIs, we calculated 95% prediction intervals (PIs) to account for the uncertainty of the effects expected in future similar studies [42–44]. Exploratory subgroup comparisons and meta-regressions were calculated for categorical (i.e., participant training status, type of SS, SS intensity, comparator, and type of control group/contralateral leg) and continuous (i.e., percent of female individuals in the sample, mean age, mean time under stretching per exercise, time under stretching per session, weekly time under stretching, total time under stretching, number of repetitions per session, number of different stretching exercises per session, weekly session frequency, and intervention period) variables, respectively.

To reduce dichotomization, we primarily focused on the point estimate with the greatest emphasis on the lower to upper limits of the CI estimates [45–47] and as a secondary source for evidence consulted *p* values.^3^*I*^2^ statistics were calculated and reported [48], with *I*^2^ statistics being calculated for the overall model, as well as to account for within- and between-study variance [49]. Heterogeneity is indicated by *I*^2^ values as follows: 0–40% no heterogeneity, 30–60% moderate heterogeneity, 50–90% substantial heterogeneity, and 75–100% considerable heterogeneity [50]. Of note, as pre-post correlations are rarely reported for within- or between-subject effects, a range of correlation coefficients was adopted (*r* = 0.5, 0.7, and 0.9) to examine the sensitivity of the results to these values. As the results were relatively insensitive to this range, they are reported for *r* = 0.7.

## Results

### Study Characteristics

**Fig. 1:**
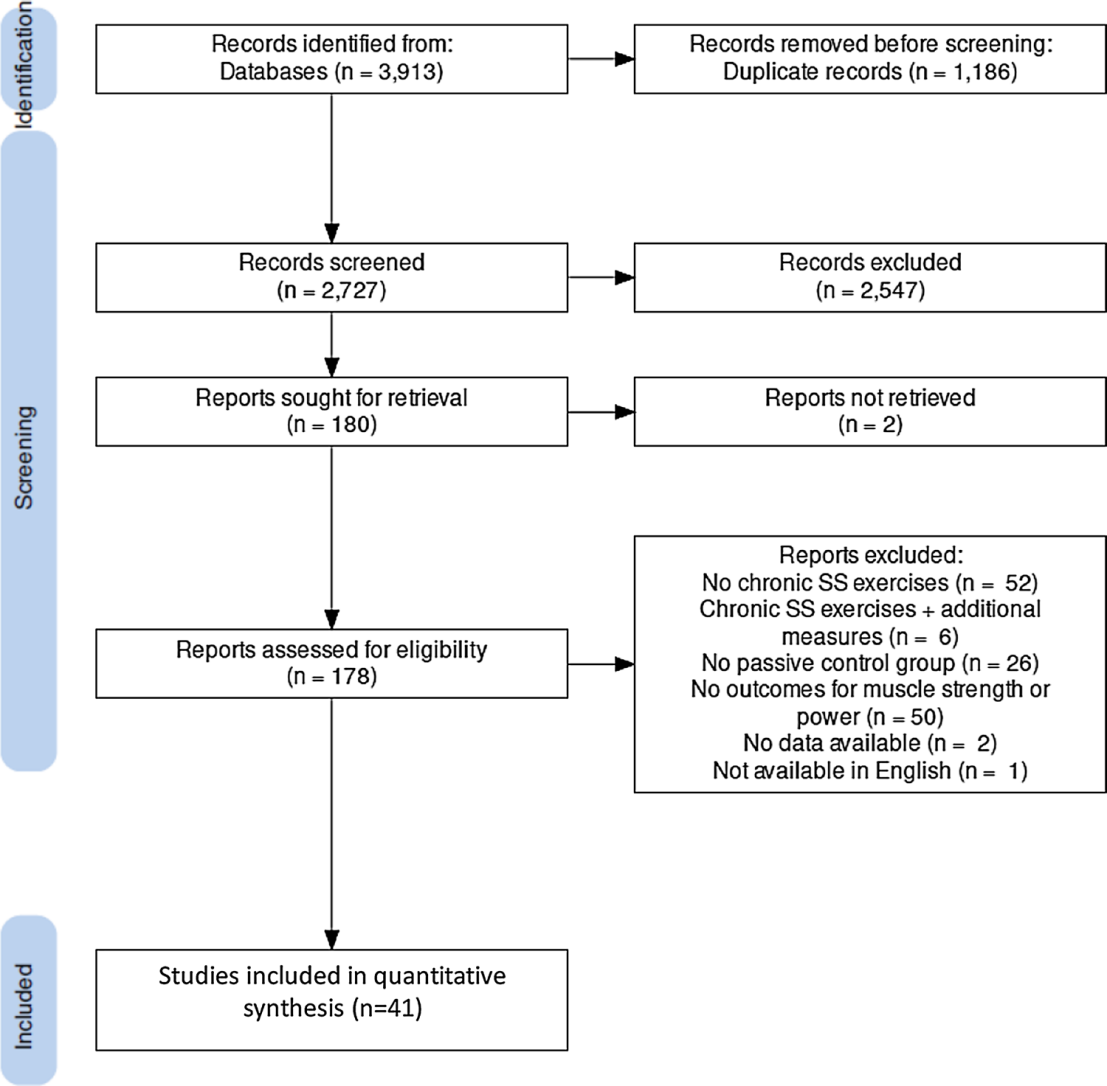
Flow chart illustrating the different stages of search and study selection. *SS* static stretching

The literature search identified 3835 studies and snowball searches added 78. After the removal of duplicates and screening of titles, abstracts, and full texts, a total of 41 studies were eligible for inclusion [7, 15–20, 28, 51–84]. Details of the search and the review of studies are presented in the flow chart (Fig. 1). The total number of participants across all included studies was 1178 (median 24, range 8–80). Of the 41 included studies, 33 assessed measures of muscle strength, 20 investigated changes in muscle power, while flexibility changes were investigated by 33 studies. With regard to participants’ training status, eight studies included sedentary individuals, 21 studies included recreationally active individuals, four examined trained athletes, one study investigated the chronic effects of SS exercises in highly trained athletes, and seven studies did not report this information. Regarding the type of SS training, 22 studies examined the implementation of passive SS, while 14 studies evaluated active SS exercises. Two studies mixed active and passive SS exercises in their interventions and six studies did not provide sufficient information to allow the classification of the SS intervention. With respect to the intensity of the applied SS, 14 studies implemented SS exercises performed below the point of discomfort (i.e., no pain), six studies used exercises performed at the point of discomfort (i.e., moderate pain), 12 studies included exercises carried out above the point of discomfort (i.e., severe pain), and ten studies did not provide sufficient information to facilitate such a classification. Regarding the comparator, 30 studies used a between-subjects design and 13 studies used a within-subjects study design. Five studies investigated female participants, 20 analyzed male participants, 16 included mixed groups, and one did not report this information. The median mean age was 22 years (range 9.7–88.8, missing: 0), the median of the mean time under stretching per exercise was 30 s (range 2–300, missing: 0), the median of the mean number of different SS exercises per session was one (range 0–15, missing: 3), the median of the mean number of repetitions per session was four (range 1–30, missing: 0), the median weekly session frequency was three (range 2–14, missing: 0), and the median intervention period was 6 weeks (range 2–24, missing: 0). Regarding the methodological quality of the included studies, PEDro scale scores ranged from 3 to 5 for studies using a within-subject design (median 5) and from 3 to 7 for studies using a between-subject design (median 4). The achieved PEDro scale percent ranged from 42.9 to 100% with a median score of 57.1%. Full details of the included studies can be seen in Tables 1 and 2.

**Table 1:** Characteristics of the included studies

Study	Study details^a^	Participant details^b^	Intervention details^c^	PEDro scale score (%)
Abdel-Aziem et al. [57]	Between-subject; 75 (50|25)	Recreationally active; 0; 22.1	Passive; 30-5-1-10-6; moderate pain	3 (42.9)
		Recreationally active; 0; 22.3	Passive; 30-5-1-10-6; moderate pain	
Akagi et al. [58]	Within-subject; 19	Recreationally active; 0; 23.7	Passive; 120-3-1-6-5; no pain	5 (83.3)
Barbosa et al. [19]	Between-subject; 45 (30|15)	Recreationally active; 0; 21.4	Active; 30-3-1-10-3; moderate pain	7 (100.0)
Batista et al. [59]	Within-subject; 12	Recreationally active; 100; 68.3	Active; 60-7-1-2-4; no pain	3 (50.0)
Bazett-Jones et al. [16]	Between-subject; 21 (10|11)	Highly trained; 100; 18.6	Active; 45-4-1-4-3; no pain	4 (57.1)
			Active; 45-4-1-4-6; no pain	
Berenbaum et al. [82]	Between-subject; 22 (17|5)	Recreationally active; 36; 21.4	Active; 30-4-2-3-3; no pain	3 (42.9)
Blazevich et al. [18]	Between-subject; 23 (14|9)	NA; 0; 18.6	Passive; 30-3-1-14-3; moderate pain	5 (71.4)
Brusco et al. [20]	Within-subject; 10	NA; 0; 24.4	Passive; 60-8-1-2-6; severe pain	3 (50.0)
Brusco et al. [60]	Within-subject; 13	NA; 0; 23.6	Passive; 60-8-1-2-6; severe pain	4 (66.7)
Caldwell et al. [84]	Between-subject; 30 (20|10)	Recreationally active; 60; 20.9	Active; 30-3-2-14-2; severe pain	5 (71.4)
		Recreationally active; 60; 22.0	Active; 30-3-2-7-2; severe pain	
	Within-subject; 20	Recreationally active; 60; 20.5	Active; 30-3-2-14-2; severe pain	5 (83.3)
		Recreationally active; 60; 22.6	Active; 30-3-2-7-2; severe pain	
Chen et al. [61]	Between-subject; 30 (20|10)	Sedentary; 0; 22.0	Active; 23-17-1-3-8; no pain	4 (57.1)
Chen et al. [62]	Between-subject; 30 (20|10)	Sedentary; 0; 20.8	Active; 30-30-1-3-8; no pain	4 (57.1)
Donti et al. [63]	Between-subject; 30 (19|11)	Trained; 100; 9.7	Passive; 30-3-1-3-3; severe pain	4 (57.1)
			Passive; 30-3-1-3-6; severe pain	
			Passive; 30-3-1-3-9; severe pain	
			Passive; 90-1-1-3-3; severe pain	
			Passive; 90-1-1-3-6; severe pain	
			Passive; 90-1-1-3-9; severe pain	
			Passive; 60-2-1-3-3; severe pain	
			Passive; 60-2-1-3-6; severe pain	
			Passive; 60-2-1-3-9; severe pain	
eLima et al. [64]	Between-subject; 23 (12|11)	Recreationally active; 0; 19.1	Active; 30-3-1-3-8; severe pain	4 (57.1)
Guissard et al. [65]	Within-subject; 12	NA; 33; 28.0	Passive; 30-5-4-5-6; severe pain	3 (50.0)
Gunaydin et al. [51]	Between-subject; 56 (42|14)	Sedentary; 45; 22.5	Mixed; 2-10-1-3-6; NA	3 (42.9)
		Sedentary; 45; 23.2	Passive; 15-10-1-3-6; NA	
Ikeda et al. [66]	Between-subject; 25 (12|13)	Recreationally active; 0; 22.0	Active; 30-6-1-3-6; moderate pain	3 (42.9)
Kokkonen et al. [7]	Between-subject; 38 (19|19)	Sedentary; 58; 20.5	Mixed; 15-3-15-3-10; NA	5 (71.4)
Konrad et al. [52]	Between-subject; 41 (20|21)	NA; 29; 23.1	Passive; 30-4-1-5-6; moderate pain	3 (42.9)
Kubo et al. [67]	Within-subject; 8	Recreationally active; 0; 24.6	Passive; 45-5-1-14-3; NA	4 (66.7)
LaRoche et al. [68]	Between-subject; 29 (19|10)	Recreationally active; 0; 33.2	Active; 30-10-1-3-4; no pain	4 (57.1)
Longo et al. [69]	Between-subject; 30 (15|15)	Recreationally active; 40; 22.7	Passive; 45-5-2-5-6; severe pain	5 (71.4)
			Passive; 45-5-2-5-12; severe pain	
Marshall et al. [70]	Between-subject; 22 (11|11)	Recreationally active; 36; 22.7	Passive; 30-3-4-5-4; NA	5 (71.4)
Meliggas et al. [71]	Between-subject; 42 (30|12)	NA; 0; 13.1	NA; 10-3-NA-3-8; NA	3 (42.9)
Minshull et al. [72]	Within-subject; 18	Recreationally active; 0; 20.7	Passive; 20-3-1-3-8; NA	4 (66.7)
Mizuno [73]	Between-subject; 20 (11|9)	Recreationally active; 38; 18.7	Passive; 30-4-1-3-8; no pain	4 (57.1)
Moltubakk et al. [74]	Within-subject; 26	Recreationally active; 61; 22.0	Active; 60-4-1-7-8; no pain	5 (83.3)
			Active; 60-4-1-7-16; no pain	
			Active; 60-4-1-7-24; no pain	
			Active; 60-4-0-7-24; no pain	
Morton et al. [75]	Between-subject; 24 (12|12)	Recreationally active; 29; 21.9	NA; 29-2-6-4-5; NA	3 (42.9)
			NA; 29-2-3-4-5; NA	
Nakamura et al. [53]	Between-subject; 40 (27|13)	Recreationally active; 0; 20.8	Passive; 30-3-1-4-4; no pain	5 (71.4)
		Recreationally active; 0; 21.6	Passive; 30-3-1-4-4; moderate pain	
Nakao et al. [76]	Between-subject; 30 (15|15)	Recreationally active; 0; 22.7	Passive; 300-1-1-3-4; no pain	4 (57.1)
Nelson et al. [15]	Between-subject; 25 (13|12)	Sedentary; 52; 23.3	Active; 30-4-1-3-10; severe pain	4 (57.1)
	Within-subject; 13	Sedentary; 54; 24.5	Active; 30-4-1-3-10; severe pain	3 (50.0)
Nóbrega et al. [77]	Between-subject; 43 (20|23)	Sedentary; 35; 21.0	Passive; 30-3-NA-2-12; no pain	4 (57.1)
Panidi et al. [54]	Within-subject; 21	Trained; 100; 13.5	Passive; 64-2-6-5-12; severe pain	5 (83.3)
Ross et al. [28]	Within-subject; 10	Recreationally active; 40; 20.3	Active; 30-5-1-7-2; no pain	4 (66.7)
Sermaxhaj et al. [78]	Between-subject; 24 (12|12)	Trained; NA; 13.9	NA; 20-1-11-3-16; NA	3 (42.9)
Simão et al. [17]	Between-subject; 80 (40|40)	Sedentary; 100; 34.0	NA; 38-4-NA-3-16; no pain	5 (71.4)
Simpson et al. [55]	Between-subject; 21 (11|10)	NA; 0; 22.0	Passive; 180-1-1-5-3; NA	4 (57.1)
			Passive; 180-1-1-5-6; NA	
Stanziano et al. [79]	Between-subject; 17 (9|8)	Sedentary; 76; 88.8	Active; 4-10-6-2-8; NA	4 (57.1)
			Active; 4-10-4-2-8; NA	
Wilson et al. [56]	Between-subject; 16 (9|7)	Trained; 0; 26.2	Passive; 16-12-4-2-8; severe pain	5 (71.4)
Yahata et al. [80]	Within-subject; 16	Recreationally active; 0; 21.4	Passive; 60-6-1-2-5; severe pain	4 (66.7)
Yuktasir et al. [81]	Between-subject; 28 (19|9)	Recreationally active; 0; 21.8	Passive; 30-4-1-4-6; severe pain	4 (57.1)

*NA* Not available, *PEDro* Physiotherapy Evidence Database

^a^Study details are presented as comparator (within subject/between subject), and *N* (intervention|control)

^b^Participant details are presented as participant training status, percentage of female individuals in the sample (%), and mean age (years)

^c^Intervention details are presented as type of static stretching (active/passive/mixed), mean time under stretching per exercise (s), number of repetitions per exercise (*n*), number of different stretching exercises per session (*n*), weekly session frequency (*n*), intervention period (weeks), stretching intensity (no/moderate/severe pain)

**Table 2:** Details of the static stretching programs across the included studies

Study	Stretched muscles [overall number of stretching exercises]	Measure	Test: joint [number of specific stretching exercises per session]
Abdel-Aziem et al. [57]	Plantar flexor [1]	Muscle strength	MVC concentric peak torque—plantar flexor [1]
			MVC eccentric peak torque—plantar flexor [1]
		Flexibility	ROM—dorsiflexion [1]
Akagi et al. [58]	Plantar flexor [1]	Muscle strength	MVC isometric peak torque—plantar flexor [1]
		Flexibility	ROM—plantar flexor [1]
Barbosa et al. [19]	Hamstring [1]	Muscle strength	MVC eccentric peak torque—knee flexor [1]
		Muscle power	Triple hop [1]
Batista et al. [59]	Knee extensor [1]	Muscle strength	MVC concentric peak torque—knee extensor [1]
			MVC concentric peak torque—knee flexor [1]
			MVC eccentric peak torque—knee extensor [1]
			MVC eccentric peak torque—knee flexor [1]
			MVC isometric peak torque—knee extensor [1]
			MVC isometric peak torque—knee flexor [1]
		Flexibility	ROM—knee extension [1]
Bazett-Jones et al. [16]	Hamstring [1]	Muscle power	Vertical jump [1]
		Flexibility	ROM—knee extension [1]
Berenbaum et al. [82]	Hamstring, quadriceps [2]	Muscle power	Horizontal jump [2]
			Vertical jump [2]
		Flexibility	ROM—knee extension [2]
			Sit and reach [2]
Blazevich et al. [18]	Plantar flexor [1]	Muscle strength	MVC peak torque—plantar flexor [1]
		Muscle power	RFD—plantar flexor [1]
		Flexibility	ROM—plantar flexion [1]
Brusco et al. [20]	Hamstring [1]	Muscle strength	MVC dynamic peak torque—knee flexor [1]
			MVC isometric peak torque—knee flexor [1]
			MVC passive peak torque—knee flexor [1]
		Flexibility	ROM [1]
Brusco et al. [60]	Hamstring [1]	Muscle strength	MVC dynamic peak torque—knee extensor [1]
		Flexibility	ROM—hip flexion [1]
			ROM—knee extension [1]
Caldwell et al. [84]	Hamstring, quadriceps [2]	Muscle strength	MVC isometric peak torque—hamstring [2]
			MVC isometric peak torque—quadriceps [2]
		Muscle power	Drop jump [2]
		Flexibility	ROM—hip flexion [2]
Chen et al. [61]	Hip extensor [1]	Muscle strength	MVC isometric peak torque—hip extensor [1]
		Flexibility	ROM—hip [1]
Chen et al. [62]	Hamstring [1]	Muscle strength	MVC concentric peak torque—hip extensor [1]
			MVC concentric peak torque—hip flexor [1]
		Flexibility	ROM—hip [1]
Donti et al. [63]	Quadriceps [1]	Muscle power	Countermovement jump [1]
		Flexibility	ROM—hip extension [1]
e Lima et al. [64]	Knee extensor, knee flexor [2]	Muscle strength	MVC isometric peak torque—knee extensor [1]
			MVC isometric peak torque—knee flexor [1]
		Flexibility	ROM—knee extension [1]
			ROM—knee flexion [1]
Guissard et al. [65]	Calf [4]	Muscle strength	MVC force—plantar flexor [4]
		Muscle power	RFD—plantar flexor [4]
		Flexibility	ROM—dorsiflexion [4]
Gunaydin et al. [51]	Hamstring [1]	Muscle power	Vertical jump [1]
		Flexibility	ROM—knee extension [1]
Ikeda et al. [66]	Knee extensor [1]	Muscle strength	MVC isometric peak torque—knee extensor [1]
		Muscle power	Countermovement jump [1]
			Rebound jump [1]
			RFD—leg extension [1]
			Squat jump [1]
		Flexibility	ROM—knee flexion [1]
Kokkonen et al. [7]	Hamstring, quadriceps, calf [15]	Muscle strength	1RM—knee extensor [15]
			1RM—knee flexor [15]
		Muscle power	Standing long jump [15]
			Vertical jump [15]
		Flexibility	Sit and reach [15]
Konrad et al. [52]	Plantar flexor [1]	Muscle strength	MVC isometric peak torque—plantar extensor [1]
		Flexibility	ROM—dorsiflexion [1]
Kubo et al. [67]	Calf [1]	Muscle strength	MVC isometric peak torque—plantar extensor [1]
LaRoche et al. [68]	Hip extensors [1]	Muscle strength	MVC isometric peak torque—hip extension [1]
			Work—hip extension [1]
		Muscle power	RFD—hip extension [1]
Longo et al. [69]	Plantar flexor [2]	Muscle strength	MVC isometric peak torque—plantar flexor [2]
		Muscle power	RFD [2]
		Flexibility	ROM—dorsiflexion [2]
Marshall et al. [70]	Hamstring, hip flexor, gluteal (2) [4]	Muscle strength	MVC isometric peak torque—hip extensor [4]
		Flexibility	ROM—hip flexion [4]
Meliggas et al. [71]	Lower extremities [NA]	Muscle power	Drop jump—20 cm [NA]
			Standing long jump [NA]
		Flexibility	ROM—hip abduction [NA]
			ROM—hip extension [NA]
			ROM—hip flexion [NA]
			ROM—knee flexion [NA]
Minshull et al. [72]	Hamstring [1]	Muscle strength	MVC isometric peak torque—knee flexor [1]
		Flexibility	ROM—hip [1]
Mizuno [73]	Calf [1]	Muscle strength	1RM—calf rise [1]
		Flexibility	ROM—dorsiflexion [1]
Moltubakk et al. [74]	Plantar flexor [1]	Muscle strength	MVC concentric peak torque—30°/s [1]
			MVC concentric peak torque—45°/s [1]
			MVC concentric peak torque—60°/s [1]
			MVC concentric peak torque—90°/s [1]
			MVC concentric peak torque—dorsi 30°/s [1]
			MVC concentric work—30°/s [1]
			MVC concentric work—45°/s [1]
			MVC concentric work—60°/s [1]
			MVC concentric work—90°/s [1]
			MVC concentric work—dorsi 30°/s [1]
			MVC isometric peak torque—-10° [0]
			MVC isometric peak torque—-15° [0]
			MVC isometric peak torque—-5° [1]
			MVC isometric peak torque—0° [1]
			MVC isometric peak torque—10° [1]
		Flexibility	ROM—ankle [1]
Morton et al. [75]	Piriformis, quadriceps, groin, hip flexor, hamstring, pectoralis, deltoid, triceps [9]	Muscle strength	MVC peak torque—knee extensor [6]
			MVC peak torque—knee flexor [6]
		Flexibility	ROM—hip extension [6]
			ROM—hip flexion [6]
			ROM—knee extension [6]
			ROM—shoulder extension [3]
Nakamura et al. [53]	Plantar flexor [1]	Muscle strength	MVC concentric peak torque—plantar flexor [1]
			MVC isometric peak torque—plantar flexor [1]
		Muscle power	Drop jump—20 cm [1]
		Flexibility	ROM—dorsiflexion [1]
Nakao et al. [76]	Hamstring [1]	Muscle strength	MVC isokinetic peak torque—knee flexor [1]
Nelson et al. [15]	Calf [1]	Muscle strength	1RM—calf rise [1]
		Flexibility	ROM—dorsiflexion [1]
Nóbrega et al. [77]	Upper limbs, lower limbs, shoulder, hip, trunk [NA]	Muscle strength	1RM—bench press [NA]
			1RM—handgrip [NA]
			1RM—leg press [NA]
Panidi et al. [54]	Plantar flexor [6]	Muscle power	Countermovement jump [6]
		Flexibility	ROM—dorsiflexion [6]
Ross et al. [28]	Hamstring [1]	Muscle power	Horizontal jump [1]
		Flexibility	ROM—knee extension [1]
Sermaxhaj et al. [78]	Neck, upper back, chest back, shoulder, mid-upper back, triceps, torso lateral flexor, hamstring, Achilles, quadriceps, hamstring, groin, chest [17]	Muscle strength	MVC isokinetic peak torque—knee extensor [11]
			MVC isokinetic peak torque—knee flexor [11]
Simão et al. [17]	Upper body, lower body, shoulders, hips, trunk [NA]	Muscle strength	10RM—bench press [NA]
			10RM—leg press [NA]
		Flexibility	Sit and reach [NA]
Simpson et al. [55]	Plantar flexor [1]	Muscle strength	MVC isometric peak torque—plantar flexor [1]
Stanziano et al. [79]	Shoulder flexor/abductor, shoulder hyperextensor, hip hyperextensor, hip abductor, shoulder hyperflexor, lateral trunk flexor, shoulder adductor, trunk rotator, trunk/hip flexor, plantar flexor [10]	Muscle strength	30-s arm curl [6]
			Chair stand test [4]
		Muscle power	Gallon jug shelf [6]
			Modified ramp power [4]
		Flexibility	Chair sit and reach [4]
			ROM—back scratch [4]
			ROM—knee extension [4]
			ROM—total body rotation [4]
			ROM [4]
Wilson et al. [56]	Shoulder, chest [4]	Muscle strength	Concentric bench press [4]
		Muscle power	Rebound bench press [4]
Yahata et al. [80]	Plantar flexor [1]	Muscle strength	MVC concentric peak torque—plantar flexor [1]
			MVC isometric peak torque—plantar flexor [1]
		Muscle power	RFD—plantar flexor [1]
Yuktasir et al. [81]	Hamstring, triceps [1]	Muscle power	Drop jump—60 cm [1]
		Flexibility	ROM—knee extension [1]

*MVC* maximum voluntary contraction, *NA* Not available, *RFD* rate of force development, *RM* repetition maximum, *ROM* range of motion

### Main Models: All Effects

**Fig. 2:**
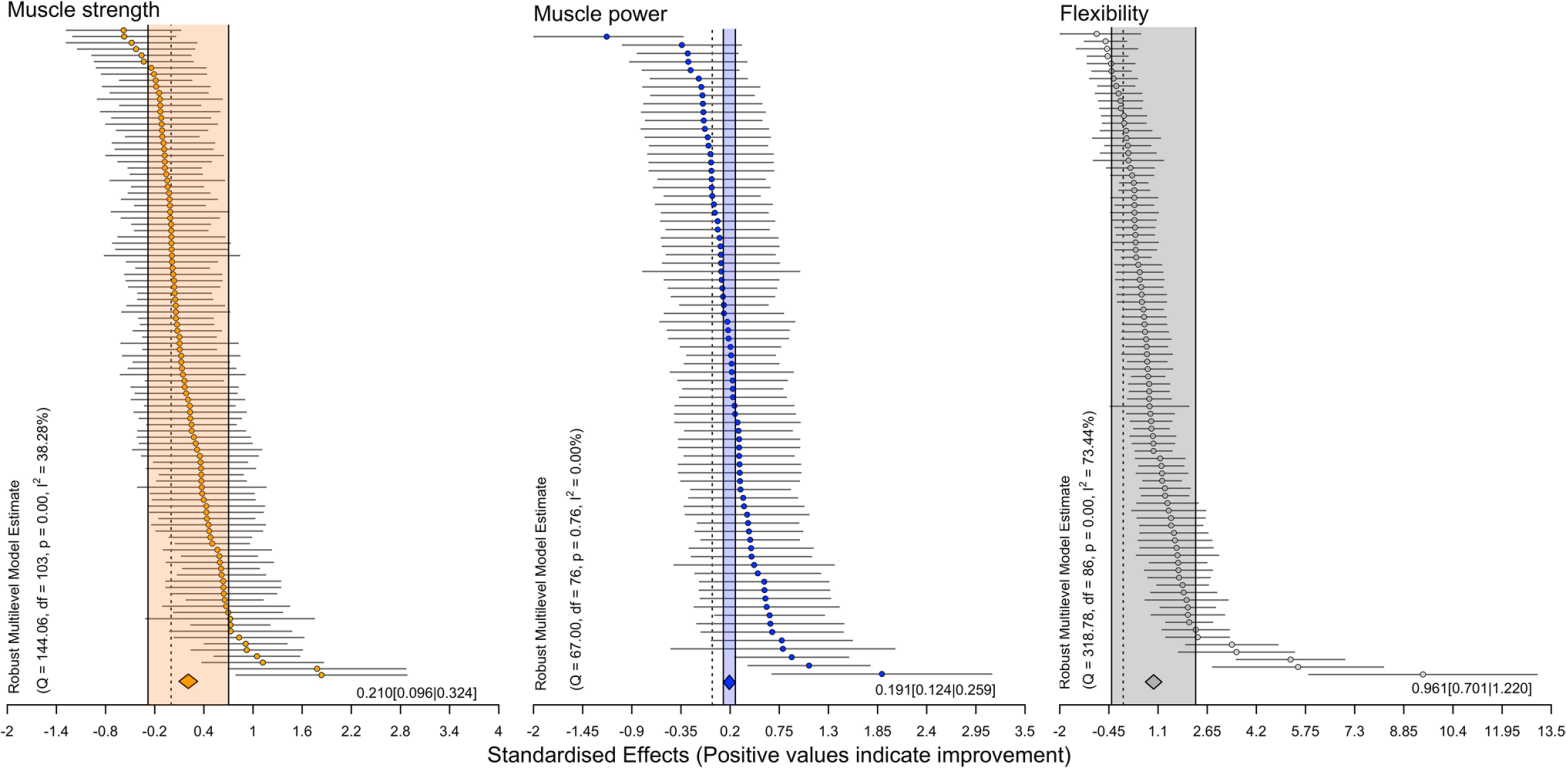
Ordered caterpillar plot with prediction intervals of all effects for muscle strength, muscle power, and flexibility. Positive values indicate chronic static stretching exercise-related improvements in muscle strength, muscle power, and flexibility. The highlighted space between the two vertical lines indicates the 95% prediction interval

The main model for muscle strength (103 effect sizes across 33 clusters [median 2, range 1–10 outcomes per cluster]) revealed trivial-to-small effects with a small point estimate and no heterogeneity. For muscle power, the main model (72 effect sizes across 20 clusters [median 2, range 1–25 outcomes per cluster]) revealed trivial-to-small effects with a trivial point estimate and no heterogeneity. Regarding flexibility, the main model (78 effect sizes across 32 clusters [median 1.5, range 1–12 outcomes per cluster]) revealed moderate-to-large effects with a large point estimate and substantial heterogeneity (Table 3, Fig. 2).

**Table 3:** Results of the main chronic effects of static stretching exercises on muscle strength, muscle power, and flexibility

Measure	Beta	CI	PI	*p* value	*I*^2a^
Muscle strength	0.210	[0.096|0.324]	[− 0.282|0.702]	0.001	38 (39, 0)
Muscle power	0.191	[0.124|0.259]	[0.124|0.259]	0.000	0 (0, 0)
Flexibility	0.961	[0.701|1.220]	[− 0.365|2.286]	0.000	73 (55, 18)

*CI* confidence interval, *PI* prediction interval

^a^Reported as *I*^2^ overall (*I*^2^ between, *I*^2^ within)

**Fig. 3:**
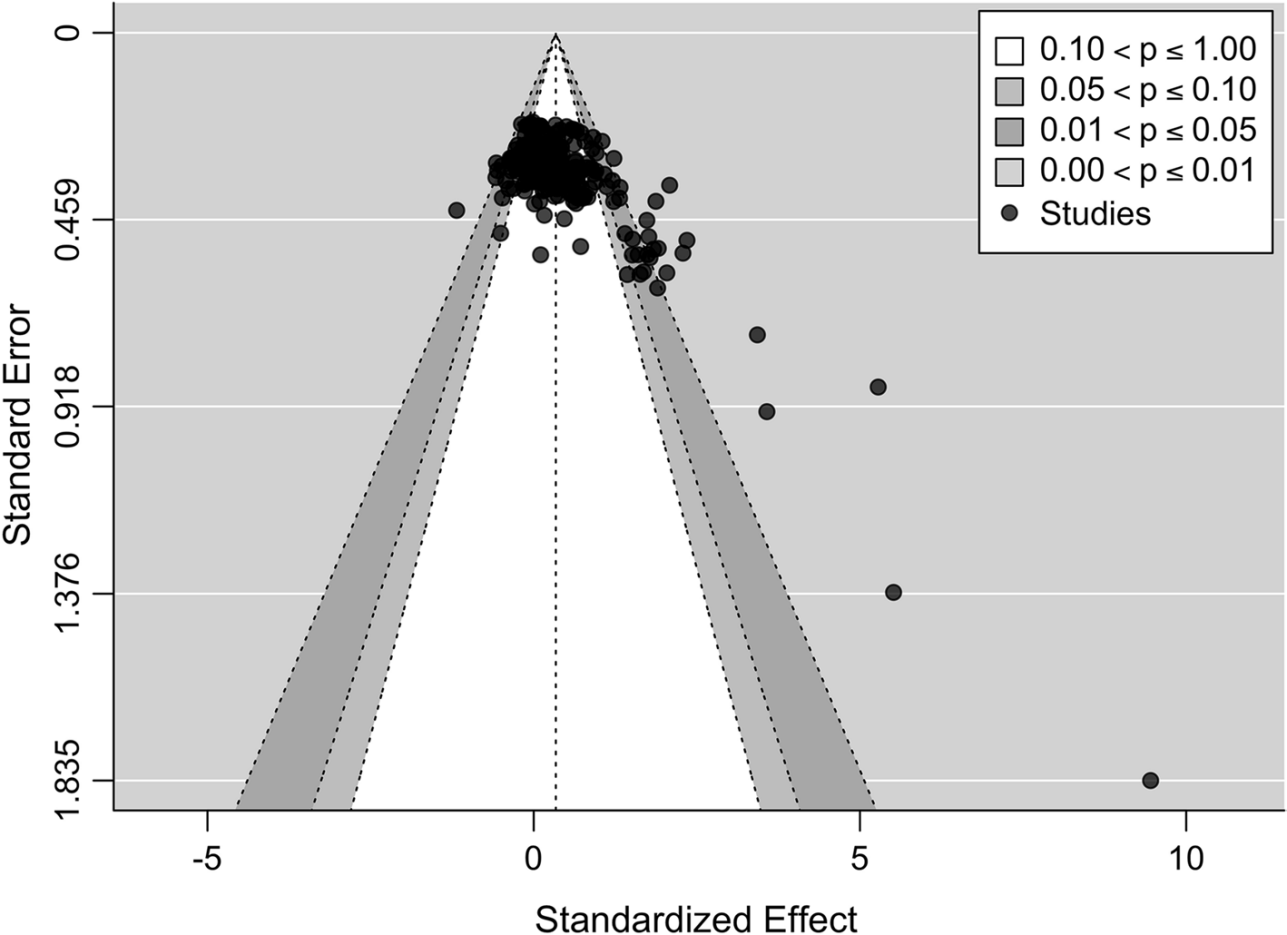
Contour-enhanced funnel plot for all effects to visualize publication bias

**Fig. 4:**
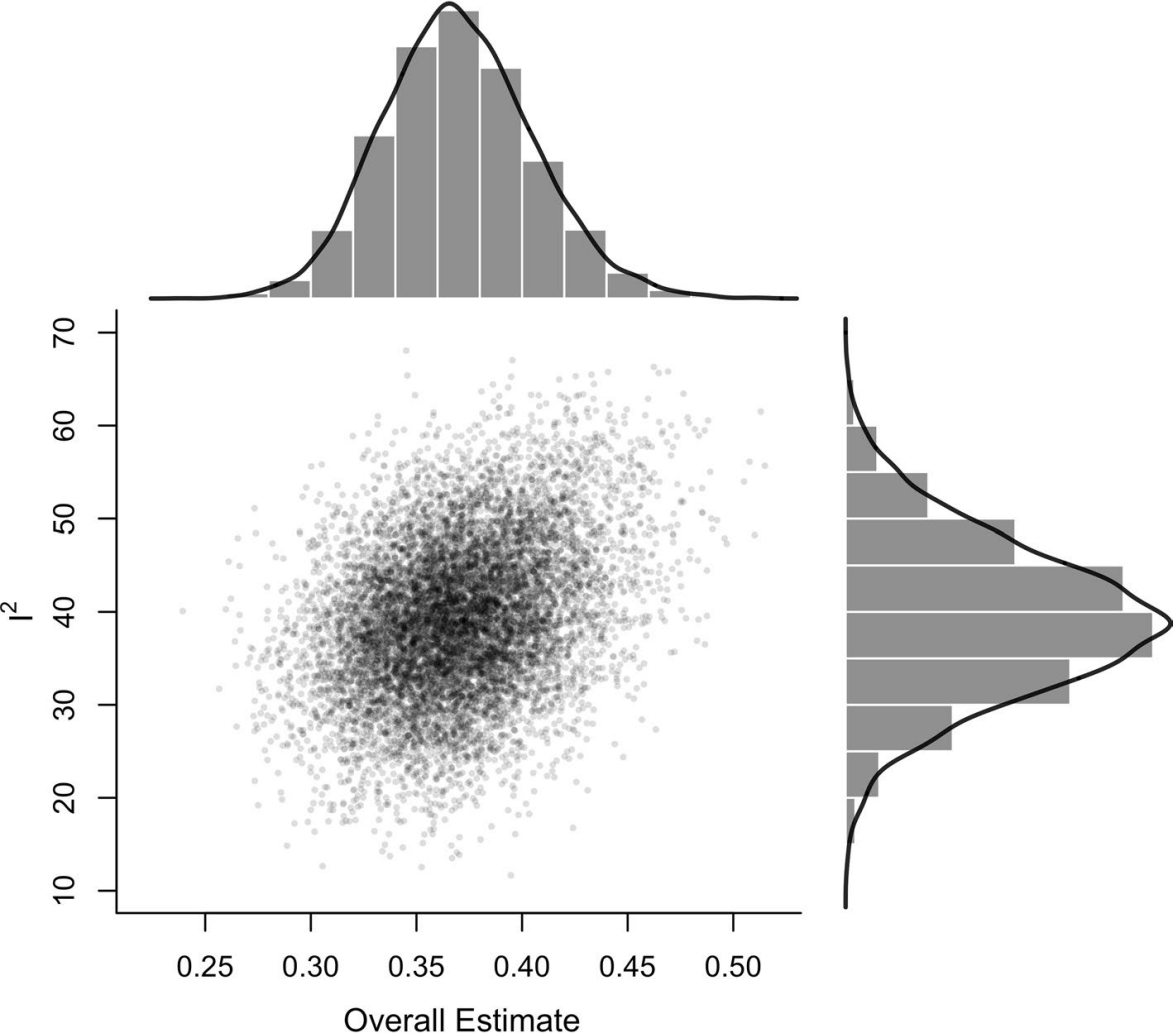
Graphical display of study heterogeneity plot for all effects

**Fig. 5:**
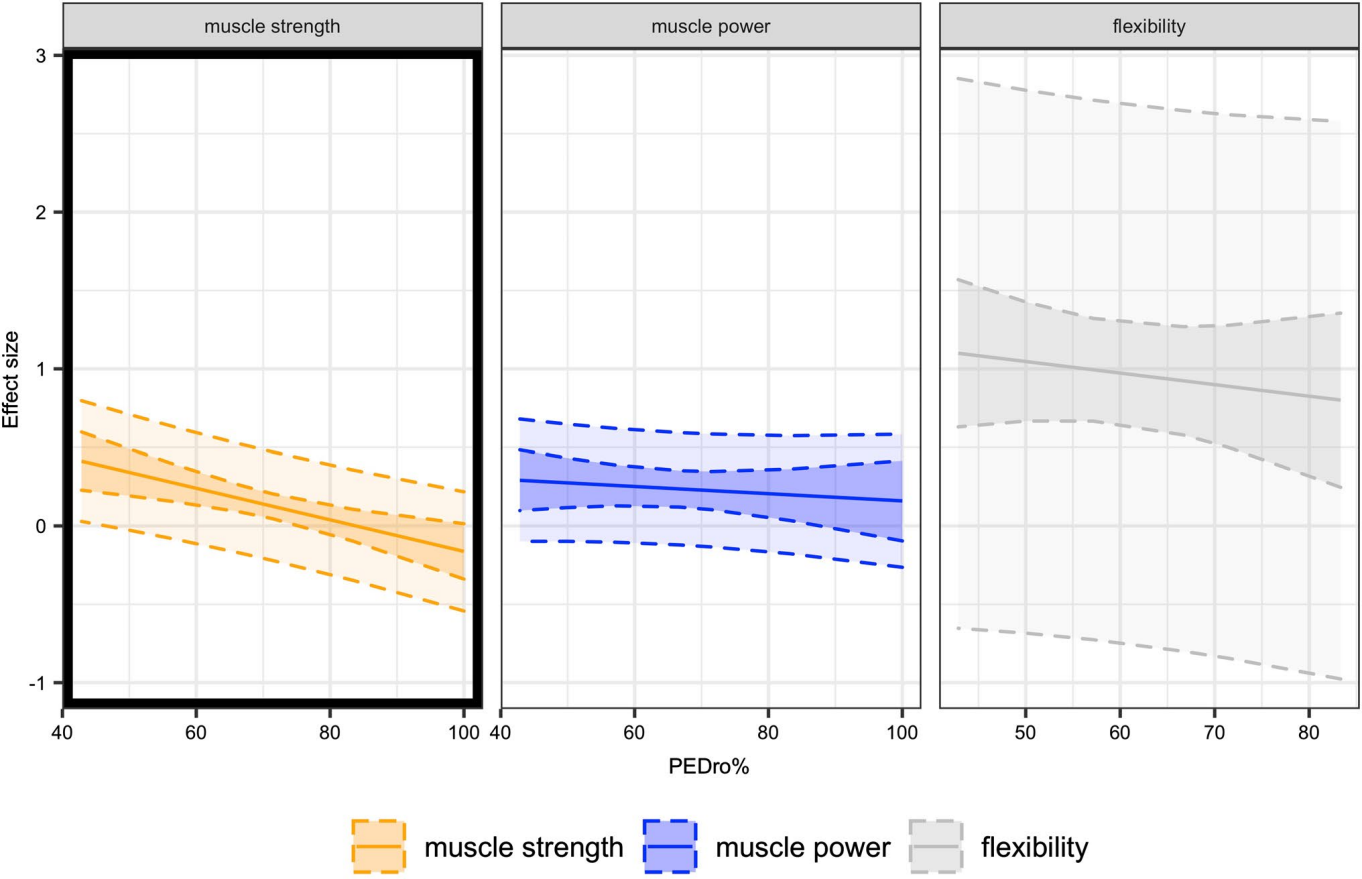
Meta-analytic regression plot of Physiotherapy Evidence Database (PEDro) scale scores (%) for muscle strength, muscle power, and flexibility. Black outlined subgroup shows meta-regression with clear effect

Visual inspection of funnel plots indicated a seemingly symmetrical distribution pattern of the effects that might be reflective of an absence of publication bias (Fig. 3). Visual inspection of the graphical display of study heterogeneity plot generally showed low levels of heterogeneity (Fig. 4). Meta-regression analysis showed that muscle strength was predicted by PEDro scale percent (SMD =  − 0.01 [95% CI − 0.02 to 0.00]; *p* = 0.002) with higher quality studies yielding smaller effect sizes. No effects were observed for muscle power and flexibility (SMD = 0.00 [95% CI − 0.01 to 0]; *p* = 0.183; SMD = 0.00 [95% CI − 0.02 to 0.02], *p* = 0.691, respectively) (Fig. 5). Subgroup analyses for study design (i.e., separate control group vs contralateral leg as a comparator) indicated no significant differences (i.e., stable effects) in muscle strength, muscle power, and flexibility. Further details can be found in the supplementary material (https://osf.io/gu9w6/).

### Subgroup Analyses

In terms of participants’ training status, subgroup analyses revealed clear small-to-large effects on muscle strength in sedentary participants with a moderate point estimate. For recreationally active participants, findings showed trivial-to-small effects with a trivial point estimate. However, results for trained participants indicated unclear effects on muscle strength with a trivial point estimate. Of note, the difference between subgroups was statistically significant. No effects were found for the participants’ training status on muscle power and flexibility.

For the type of SS exercises, the subgroup analysis revealed small-to-moderate effects on flexibility with a moderate point estimate for active SS exercises and moderate-to-large effects with a large point estimate for passive SS exercises. Of note, the difference between subgroups was statistically significant. No effects were found regarding the type of SS exercises on muscle strength and power.

**Fig. 6:**
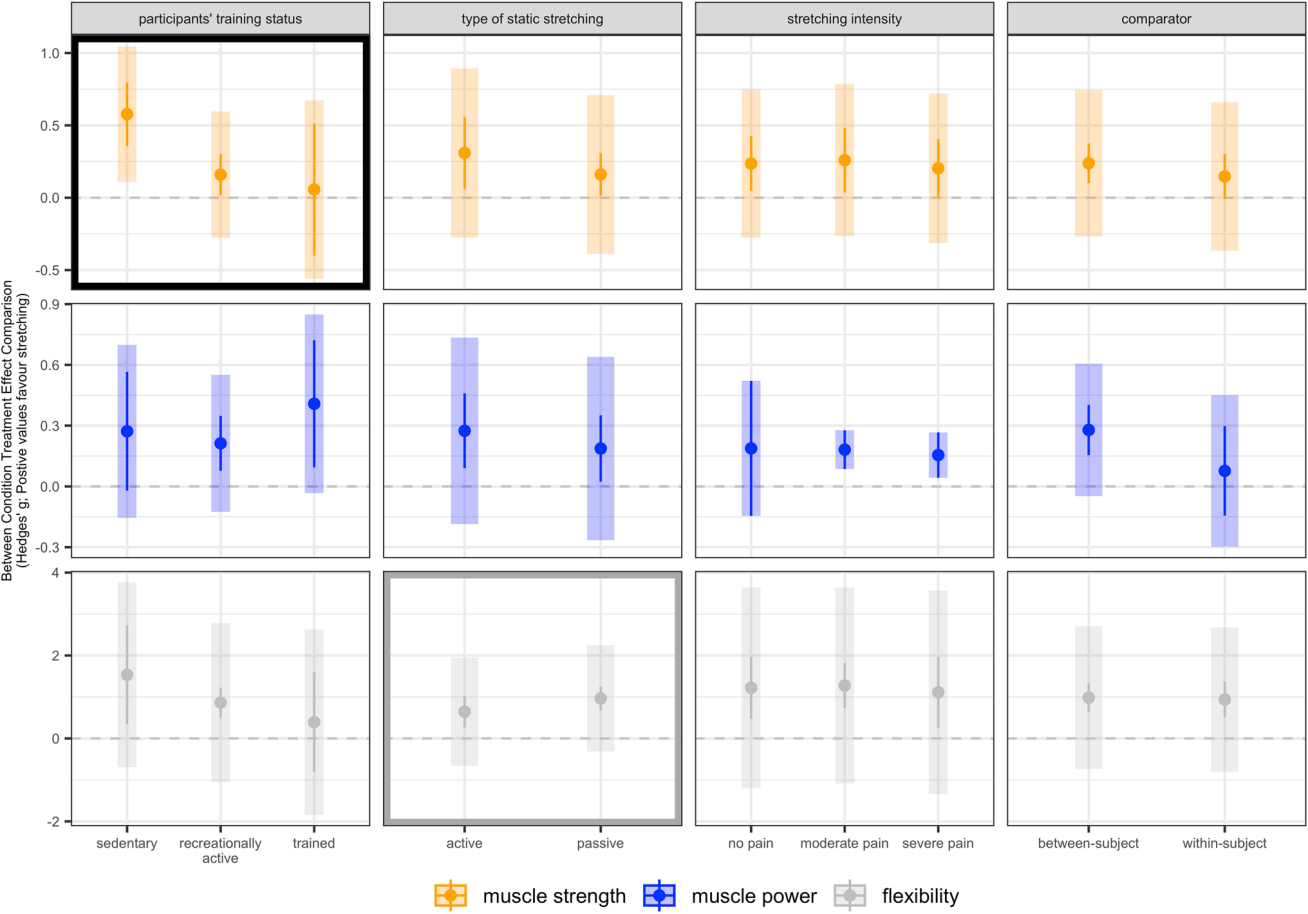
Subgroup plots of effects of chronic static stretching exercises in categorical subgroups on muscle strength, muscle power, and flexibility including prediction intervals. Black outlined plots show subgroups with clear effects and gray outlined plots show subgroups with unclear but significant effects

Further, no chronic effects for SS intensity and the type of comparator were found on muscle strength, power, and flexibility. All results of the subgroup analyses are displayed in Table 4 and Fig. 6.

**Table 4:** Results of subgroup analyses

Subgroup							
Measure	Level	Beta	CI	PI	*p* value within	*p* value between	*I*^2a^
Participants’ training status							
Muscle strength	**Sedentary**	**0.578**	**[0.358|0.798]**	**[0.108|1.048]**	**0.000**	**0.008**	**32 (32, 0)**
	**Recreationally active**	**0.161**	**[0.018|0.303]**	**[− 0.279|0.600]**	**0.029**		
	Trained	0.057	[− 0.400|0.514]	[− 0.561|0.674]	0.799		
Muscle power	Sedentary	0.255	[− 0.009|0.519]	[− 0.024|0.534]	0.057	0.920	2 (2, 0)
	Recreationally active	0.201	[0.063|0.339]	[0.036|0.366]	0.008		
	Trained	0.225	[0.037|0.412]	[0.017|0.433]	0.023		
Flexibility	Sedentary	1.476	[0.331|2.621]	[− 0.615|3.567]	0.014	0.453	83 (63, 20)
	Recreationally active	0.850	[0.493|1.206]	[− 0.936|2.635]	0.000		
	Trained	1.089	[0.648|1.531]	[− 0.715|2.894]	0.000		
Type of static stretching							
Muscle strength	Active	0.309	[0.060|0.559]	[− 0.275|0.894]	0.017	0.301	43 (43, 0)
	Passive	0.161	[0.015|0.307]	[− 0.387|0.709]	0.032		
Muscle power	Active	0.252	[0.078|0.427]	[0.078|0.427]	0.007	0.233	0 (0, 0)
	Passive	0.144	[0.083|0.206]	[0.083|0.206]	0.000		
Flexibility	Active	0.591	[0.280|0.901]	[− 0.286|1.468]	0.001	0.048	52 (16, 36)
	Passive	0.974	[0.754|1.195]	[0.125|1.823]	0.000		
Stretching intensity							
Muscle strength	No pain	0.237	[0.049|0.425]	[− 0.275|0.749]	0.016	0.922	38 (38, 0)
	Moderate pain	0.263	[0.037|0.490]	[− 0.264|0.790]	0.025		
	Severe pain	0.204	[0.003|0.405]	[− 0.313|0.721]	0.047		
Muscle power	No pain	0.167	[− 0.170|0.503]	[− 0.170|0.503]	0.304	0.934	0 (0, 0)
	Moderate pain	0.181	[0.080|0.281]	[0.080|0.281]	0.002		
	Severe pain	0.155	[0.043|0.267]	[0.043|0.267]	0.010		
Flexibility	No pain	1.224	[0.456|1.992]	[− 1.229|3.677]	0.003	0.951	90 (76, 14)
	Moderate pain	1.266	[0.727|1.806]	[− 1.125|3.657]	0.000		
	Severe pain	1.115	[0.254|1.977]	[− 1.368|3.599]	0.014		
Comparator							
Muscle strength	Between-subject	0.239	[0.100|0.378]	[− 0.272|0.750]	0.001	0.309	39 (39, 0)
	Within-subject	0.147	[− 0.007|0.302]	[− 0.368|0.662]	0.061		
Muscle power	Between-subject	0.207	[0.130|0.285]	[0.130|0.285]	0.000	0.118	0 (0, 0)
	Within-subject	0.077	[− 0.071|0.224]	[− 0.071|0.224]	0.291		
Flexibility	Between-subject	0.984	[0.682|1.286]	[− 0.467|2.436]	0.000	0.867	77 (53, 24)
	Within-subject	0.950	[0.540|1.361]	[− 0.528|2.429]	0.000		

Bold text indicates clear effects between subgroups

*CI* confidence interval, *PI* prediction interval

^a^Reported as *I*^2^ overall (*I*^2^ between, *I*^2^ within)

### Meta-regression Analyses

**Fig. 7:**
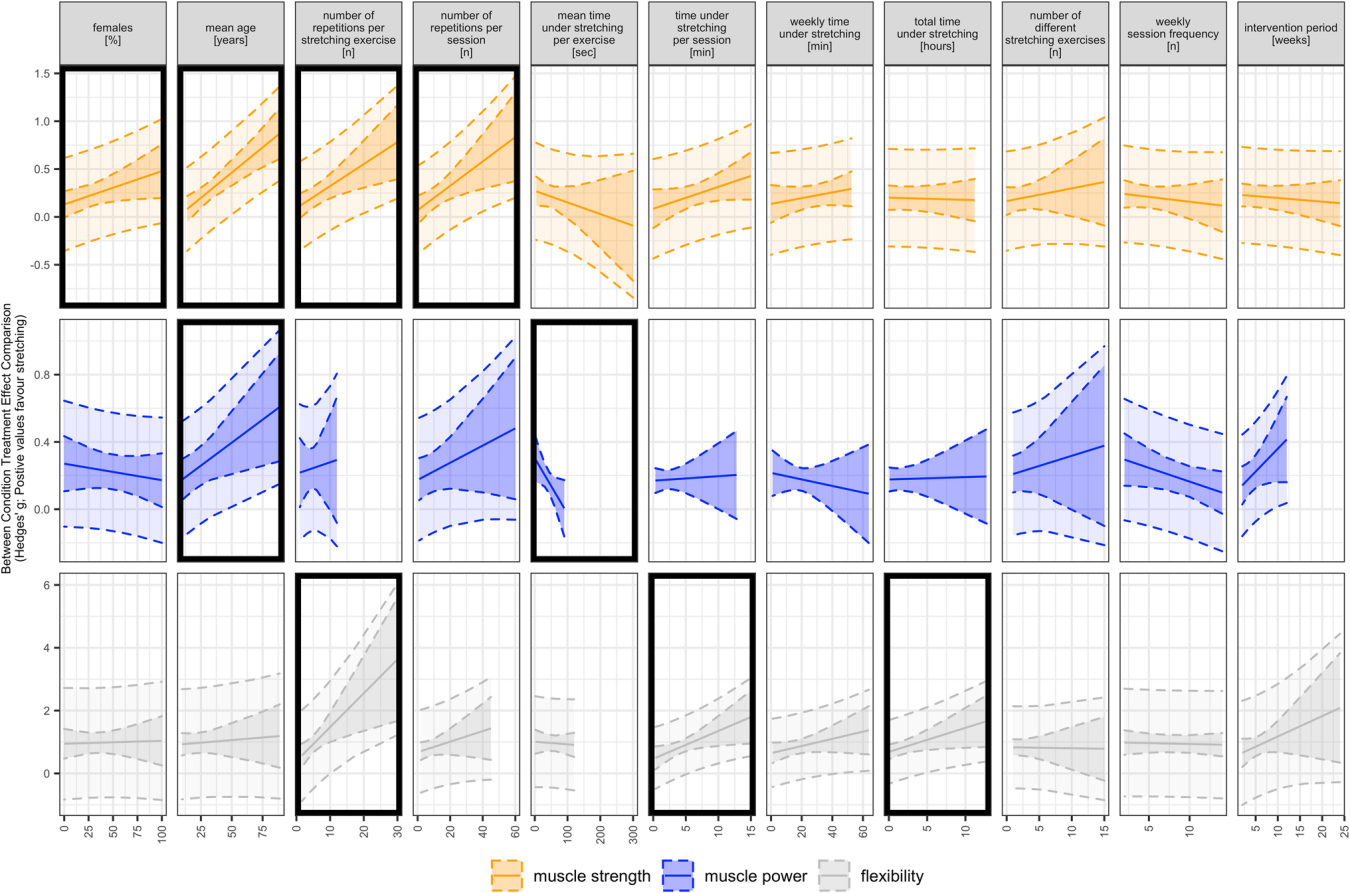
Meta-analytic plots of effects of chronic static stretching exercises in continuous subgroups on muscle strength, muscle power, and flexibility including prediction intervals. Black outlined subgroups show meta-regressions with clear effects

The meta-regression analyses showed that the chronic effects of SS exercises on muscle strength were moderated by the proportion of female individuals in the sample, with higher proportions per study associated with larger gains, participants’ mean age with older participants demonstrating larger gains, and the number of repetitions per stretching exercise and session with higher numbers associated with larger gains. Meta-regression analyses further revealed the moderating effects of the participants’ mean age on muscle power with larger gains for older participants. For flexibility, there were moderating effects of the number of repetitions per exercise with higher numbers associated with larger gains, and the time under stretching per session and in total with longer durations associated with larger benefits. All results of the meta-regression analyses are presented in Table 5 and Fig. 7.

**Table 5:** Results of the meta-regression analyses

Subgroup	Measure	Beta	CI	*p* value	*I*^2a^
Female individuals [%]					
	**Muscle strength**	**0.004**	**[0.000|0.007]**	**0.042**	**41 (41, 0)**
	Muscle power	0.000	[− 0.001|0.002]	0.884	23 (23, 0)
	Flexibility	− 0.001	[− 0.010|0.008]	0.834	91 (83, 8)
Mean age [years]					
	**Muscle strength**	**0.011**	**[0.006|0.015]**	**0.000**	**31 (31, 0)**
	**Muscle power**	**0.006**	**[0.002|0.010]**	**0.007**	**0 (0, 0)**
	Flexibility	0.002	[− 0.010|0.015]	0.697	87 (72, 15)
Number of repetitions per stretching exercise [*n*]					
	**Muscle strength**	**0.023**	**[0.008|0.038]**	**0.004**	**36 (36, 0)**
	Muscle power	0.020	[− 0.017|0.056]	0.273	0 (0, 0)
	**Flexibility**	**0.094**	**[0.019|0.169]**	**0.016**	**75 (46, 29)**
Number of repetitions per session [*n*]					
	**Muscle strength**	**0.013**	**[0.004|0.022]**	**0.008**	**36 (36, 0)**
	Muscle power	0.005	[− 0.002|0.012]	0.178	11 (11, 0)
	Flexibility	0.015	[− 0.008|0.038]	0.189	84 (68, 16)
Mean time under stretching per exercise [s]					
	Muscle strength	− 0.001	[− 0.003|0.001]	0.280	52 (52, 0)
	Muscle power	− 0.003	[− 0.006|0.000]	0.056	13 (13, 0)
	Flexibility	− 0.001	[− 0.006|0.004]	0.794	87 (73, 15)
Time under stretching per session [min]					
	Muscle strength	0.023	[− 0.003|0.050]	0.085	39 (39, 0)
	Muscle power	0.003	[− 0.022|0.027]	0.816	11 (11, 0)
	**Flexibility**	**0.090**	**[0.011|0.168]**	**0.026**	**69 (42, 27)**
Weekly time under stretching [min]					
	Muscle strength	0.003	[− 0.003|0.009]	0.297	42 (42, 0)
	Muscle power	− 0.002	[− 0.008|0.005]	0.548	0 (0,0)
	Flexibility	0.012	[− 0.004|0.028]	0.147	78 (55,24)
Total time under stretching [h]					
	Muscle strength	− 0.002	[− 0.022|0.017]	0.812	48 (48, 0)
	Muscle power	0.002	[− 0.022|0.026]	0.870	22 (22, 0)
	**Flexibility**	**0.078**	**[0.006|0.149]**	**0.034**	**84 (69, 15)**
Number of different stretching exercises [*n*]					
	Muscle strength	0.013	[− 0.022|0.048]	0.440	47 (47, 0)
	Muscle power	0.005	[− 0.010|0.020]	0.480	22 (22, 0)
	Flexibility	0.017	[− 0.023|0.058]	0.390	87 (74, 13)
Weekly session frequency [*n*]					
	Muscle strength	− 0.010	[− 0.039|0.018]	0.465	47 (47, 0)
	Muscle power	− 0.006	[− 0.017|0.005]	0.272	20 (20, 0)
	Flexibility	− 0.030	[− 0.070|0.010]	0.136	90 (75, 15)
Intervention period [weeks]					
	Muscle strength	− 0.004	[− 0.015|0.007]	0.471	46 (46, 0)
	Muscle power	0.016	[− 0.004|0.035]	0.104	0 (0, 0)
	Flexibility	0.066	[− 0.029|0.160]	0.165	76 (50, 25)

Bold text indicates regression analyses revealing clear effects

*CI* confidence interval

^a^Reported as *I*^2^ overall (*I*^2^ between, *I*^2^ within)

## Discussion

The main findings of this meta-analysis indicate that chronic SS exercises resulted in trivial-to-small improvements in muscle strength and power. For flexibility, chronic SS exercises induced moderate-to-large enhancements. Additionally, subgroup analyses showed larger effects of SS exercises on muscle strength in sedentary compared to recreationally active and trained participants, and larger effects of passive SS exercises, compared with active SS exercises, on flexibility. Furthermore, results of the meta-regression analysis for muscle strength indicated that the chronic effects of SS exercises were moderated by the percentage of female individuals in the sample with studies  including higher proportions demonstrating larger gains. Participants’ mean age, with older participants showing larger gains, and the number of repetitions per stretching exercise and session, with higher numbers associated with larger gains, were also influential moderators. For muscle power, the meta-regression analysis suggested that there were moderating effects of participants’ mean age, with larger gains for older participants. For flexibility, the meta-regression revealed moderating effects of the number of repetitions per exercise, with higher numbers associated with larger gains, and the time under stretching per session and total time under stretching with longer durations associated with larger benefits.

### Main Effects

To the authors’ knowledge, this is the first meta-analysis examining the chronic effects of SS exercises on muscle strength, muscle power, and flexibility in healthy participants. Interestingly, our findings showed beneficial effects, though trivial to small in magnitude, of SS training on muscle strength and power. These findings are in line with earlier studies on this topic [7, 14, 23, 85]. For instance, Worrell et al. [14] investigated the long-term effects of SS exercises on maximal voluntary strength of the knee flexors in healthy active young adults. With the participants undertaking 15 sessions with 20 min per session over three weeks, these researchers reported a significant increase in eccentric peak torque at 60°/s and 120°/s (∆8.5% and 13.5%, respectively), with an 11.2% increase in concentric peak torque at 120°/s. Hunter and Marshall [85] examined the effects of ten weeks of SS training on measures of muscle power (i.e., countermovement jump height) in physically active male individuals (primarily basketball and volleyball players) aged 24 years, demonstrating increased jump height (∆1.3%, compared to a non-stretching control − 0.3%).

The mechanisms underpinning the trivial-to-small gains in muscle strength and power following chronic SS exercises have yet to be established and therefore remain elusive. However, a common theory is that chronic SS exercises seem to contribute to muscle growth and hence skeletal muscle hypertrophy [54, 55, 86]. Recently, Panidi et al. [54] examined the effects of a 12-week, five times per week program of SS exercises on gastrocnemius architecture in adolescent female volleyball players. The researchers’ results indicated larger improvements in gastrocnemius cross-sectional area and fascicle length of the stretched leg as well as larger one-leg countermovement jump performance compared with the control leg. Andrade et al. [87] investigated the effects of 12 weeks of SS training on triceps surae architecture in university students. While they did not report any differences in gastrocnemius muscle thickness, they found changes in gastrocnemius medialis fascicle length in the triceps surae stretching group, with no such result observed in the control group. It is, however, important to mention that increased muscle hypertrophy following chronic SS exercises, was not consistently detected in the literature [53, 88, 89]. In a recently published narrative review on this topic, Nunes et al. [89] indicated that passive low-intensity stretching seems not to promote changes in muscle size and architecture. However, the same authors speculated that stretching with a high intensity might produce sufficient tensile strain to elicit muscle hypertrophy [89].

Albeit controversial, another potential theory is that chronic SS exercises alter the mechanical properties of the muscle–tendon unit (MTU). More specifically, there is evidence of increased MTU compliance following chronic SS exercises [90, 91], which, in turn, might allow for more efficient use of elastic energy during activities involving the stretch–shortening cycle (e.g., jumping, rebound bench press, jogging) [14, 56, 92, 93]. In this sense, the improvement in muscle power following chronic SS exercises could also be explained by the increased length of the stretched muscle, owing to an increased number of sarcomeres in series [94, 95], which in turn would improve the muscles’ contraction velocity and power [96]. However, it is worth noting that other studies did not report any changes in the mechanical properties of the MTU following chronic SS exercises [2, 97, 98], implying that this research question is still open for much discussion in future studies. Of note, although most of the 95% PI in the present study was above zero for muscle strength, which indicates that chronic SS exercises could be effective in most future studies, the interval overlaps zero and so in some upcoming studies, no effect may be apparent (Fig. 2). For muscle power, both ends of 95% PI are above zero suggesting that 95% of the future studies will find positive effects of long-term SS exercises (Fig. 2).

With the principle of training specificity in mind [99], the moderate-to-large effects of chronic SS exercises on flexibility was an expected outcome. It should be noted that most of the PI is above zero, indicating that SS training  will be effective in most future studies. However, the 95% PI does overlap zero, which means that in some future studies, specific doses of SS training might be ineffective. Several studies have shown that chronic SS exercises improve flexibility [2, 3, 52]. Two mechanisms have been suggested to explain the observed increases in joint ROM [100]. The first and most accepted theory pertains to sensory perception (i.e., sensory theory), which proposes that chronic exposure to stretching results in an increased stretch tolerance [100]. More specifically, it has been argued that the MTU can tolerate more passive tension after training owing to a modification of the subjective perception of discomfort [2, 97, 100], probably caused by adaptions at the level of nociceptive endings [52]. The second is called ‘mechanical theory’, which assumes that stretching protocols decrease joint resistance to a stretch probably because of a change in MTU mechanical properties (e.g., decrease in tissues stiffness), geometry (e.g., the addition of sarcomeres in series and increase in fascicle length), or both [100, 101]. However, the underlying mechanisms of chronic SS exercise-related flexibility adaptation are still a subject of much debate [89, 100]. Future research may provide further insights into the most prominent mechanisms.

There are substantial commonalities among training routines. While SS may induce trivial-to-small magnitude strength gains, resistance training can provide relatively greater magnitude gains. Similarly, whereas SS improves flexibility, resistance training can also improve the ROM [102, 103]. The interaction of both techniques may be necessary as athletes for example would not perform resistance training as part of their warm-up before a competition or practice, and flexibility training can be used as an alternative low-intensity strength training program, especially for seniors or individuals undergoing rehabilitation. Although the underlying mechanisms of the concomitant increase in flexibility, muscle strength, and muscle power after chronic SS exercises reported in this study still need to be explored, the current results are relevant for practitioners to set appropriate training goals.

### Subgroup Analyses

Our analysis revealed that the positive effect of chronic SS exercises on muscle strength progressively decreases with increasing training status. Specifically, chronic SS exercises result in positive and larger effects on muscle strength in sedentary as compared with recreationally active participants, while in trained participants, unclear effects were observed. The present results are additionally supported by the 95% PI. Specifically, both ends of the interval indicate that future similar studies in sedentary participants will consistently show a positive effect of chronic SS exercises on muscle strength (95% PI 0.11–1.05). However, for recreationally active and trained participants, the PIs overlapped zero (95% PI − 0.28 to 0.60 and 95% PI − 0.56 to 0.67, respectively), indicating that inconsistent findings might be expected in future studies. These findings are not surprising, as there is ample evidence that less compared to more trained participants achieved larger adaptations following training [104, 105]. The attenuated training-related adaptations in more compared to less trained individuals have been attributed to the phenomenon of a “ceiling effect”. The “ceiling effect” means that trained individuals are close to, or at, their upper limit of potential adaption to a given stimulus and therefore display limited trainability when exposed to that stimulus [106]. For example, a study investigating the effects of six weeks of SS training of the hamstring muscles in Division III women’s track and field athletes found no changes in knee ROM, 55-m sprint time, and vertical jump height in a stretching group compared to the non-stretching control group [16]. In contrast, a study investigating the effects of a ten-week calf muscle SS training in inactive undergraduate students showed improvements in a one-repetition maximum calf raise in the stretching compared with the non-stretching control group [15]. Of note, none of the included studies has directly contrasted the chronic effects of SS exercises between trained and non-trained participants, pointing to a gap in the literature. Future investigations should examine the specific mechanisms underpinning the larger benefits of chronic SS exercises in sedentary, as compared to recreationally active and trained individuals.

Additionally, a subgroup analysis revealed significantly larger effects of passive compared with active chronic SS exercises on flexibility. Active SS requires the contraction of the agonist muscles, while passive SS relies on using external forces such as gravity, applied pressure on a limb from a partner, or stretching aids such as elastic bands [1]. Our results suggest that to achieve better flexibility levels, passive SS exercises should be favored over active SS exercises. This is in agreement with the results of a study by Nishikawa et al. [84] examining the acute effects of passive versus active SS on hamstring flexibility in healthy young participants. The authors reported larger immediate effects of the former compared with the latter. Unlike our findings, results of an intervention study on the effects of 6 weeks of passive versus active SS exercises on hamstring flexibility in healthy male and female individuals aged 23 years revealed larger increases following active compared with passive SS [51]. Overall, studies comparing active with passive SS exercises are scarce and the available studies provide inconsistent findings [107, 108]. Moreover, the mechanistic aspects underlying the different effects of active or passive SS exercises on flexibility are yet to be identified.

### Meta-regression Analyses

Results of the meta-regression analyses indicated that the chronic effects of SS exercises on muscle strength are mediated by the proportion of female participants in each study, with higher proportions being associated with larger gains. A substantial body of evidence indicates sex differences in the integration of physiological systems, including the neuromuscular system, during exercise [109]. This implies that the physiological responses to equivalent dosages of exercise are different between male and female individuals [109]. Additionally, although speculative, the sex difference seems to be partly  due to the different levels of trainability and/or physical fitness. In other words, female individuals tend to be less active than male individuals and therefore display a greater potential to adapt to training than male individuals. The lower levels of physical fitness in female individuals can be attributed to the systematic exclusion of women from organized sports [110, 111] and restricted access to sports and physical activities [112]. Future investigations into the mechanisms of the long-term SS-induced strength gains should therefore take these sex differences into account. Additionally, our findings showed a moderating effect of age with larger muscle strength and power benefits in older compared with younger participants. As with male versus female individuals, the larger gains in older populations could be attributed to an age-related decline in physical activity and, therefore, physical fitness [113]. This would increase the potential to adapt to the exposed training stimulus in older participants. Moreover, the chronic effect of SS exercises on muscle strength was moderated by the number of repetitions per stretching exercise and session with a higher number resulting in larger benefits. Of note, SS exercises could be considered a form of low-intensity eccentric muscle action [114]. In this sense, the repetitive nature of such an exercise (i.e., a greater number of repetitions per stretching exercise and session) results in a distinct loading characteristic that could promote muscle strength adaptations. However, this observation is not conclusive and further investigations may still be needed to substantiate the current results.

With respect to flexibility, results of the meta-regression analyses indicated a moderating effect of the number of repetitions per exercise with higher numbers associated with larger gains. In addition, findings indicated moderating effects of the amount of time under stretching per session and the total time under stretching, with longer durations associated with larger flexibility improvements. These observations reflect the importance of SS training volume, with higher volumes promoting larger flexibility gains. While evidence around the number of repetitions is scarce, the time under stretching has been more thoroughly investigated in the literature. For example, a meta-analysis investigating the effects of different stretching types (i.e., ballistic, proprioceptive neuromuscular facilitation, and static [active, passive, and unspecified]) on joint ROM showed a weekly time under stretching of ≥ 5 min induced larger improvements compared to < 5 min with no effect of time under stretching per session [115]. Another meta-analysis assessing the chronic effect of SS exercises on ankle dorsiflexion’s ROM showed no difference between the total time under stretching of < 3000 s, 3000–5000 s, and > 5000 s [116]. In a study investigating the effects of different SS volumes, Bandy and colleagues [117] compared four different stretching protocols (i.e., 3 * 60 s, 3 * 30 s, 1 * 60 s, and 1 * 30 s) implemented four times per week for 6 weeks compared to a passive control group. While the authors found all stretching protocols induced improvements in knee extension ROM, compared with a passive control, they detected no differences between the different protocols. Similarly, Cipriane et al. [118] investigated the effects of four different hamstring SS protocols (i.e., twice daily, once daily, twice every second day, and once every second day for 1 min) for four weeks and found similar improvements for hip ROM following all protocols. Overall, our findings advance the general trend in the literature that larger SS training volumes induce larger gains in flexibility. However, further investigations focusing on the interactions between the time under SS and the number of repetitions could allow a more refined understanding of the effect on flexibility.

### Future Research Perspectives

The mechanisms underpinning chronic SS exercise-induced muscle strength and power improvements are not yet well understood and are rather speculative. More particularly, the mechanisms underlying the concomitant changes in flexibility, muscle strength, and muscle power are not known and therefore require further investigation. Thus, future studies exploring the mechanisms by which chronic SS exercises promote muscle strength and power increments are needed. Additionally, none of the existing studies directly contrasted the chronic effects of SS exercises on muscle strength and power between male and female individuals, trained and sedentary, as well as older and younger adults. Therefore, future studies should be conducted to investigate the mechanisms underlying the moderating effects of sex, training status, and age. Moreover, and based on our findings, the number of repetitions per exercise and session seems to moderate the chronic effects of SS exercises on muscle strength adaptations. However, such an outcome was derived from separate studies and could be described as indirect evidence. Therefore, further studies directly contrasting different SS training volumes (e.g., low vs high number of repetitions per exercise and session) are required to substantiate the current results.

### Limitations

This study has some limitations that must be acknowledged. The first is that moderator analyses were computed independently, ignoring any potential interaction between variables. Thus, the results of univariate analyses must be considered with caution. Additionally, a meta-regression-analysis regarding study quality revealed that muscle strength studies of higher quality have found smaller gains. Thus, assuming higher quality studies produce effects closer to the real effect owing to a more precise and methodologically tailored approach, the effect on muscle strength indicated in the current study should be considered with caution.

## Conclusions

This systematic review with a multi-level meta-analysis of 41 original studies brings forth findings with relevant practical implications. Indeed, results indicated that chronic SS exercises have the potential to improve muscle strength and power, although with a limited trivial-to-small magnitude. Additionally, as expected, our findings indicated moderate-to-large gains in flexibility following chronic SS exercises with larger effects of passive compared with active SS exercises. A subgroup analysis further indicated no evidence that SS intensity moderates the effects on muscle strength, power, or flexibility. Furthermore, results of the meta-regression analysis for muscle strength indicated that the chronic effects of SS are moderated by the proportion of female individuals in the sample with higher proportions associated with larger gains, participants’ mean age, with older participants showing larger gains, and the number of repetitions per stretching exercise and session, with higher numbers associated with greater benefits. Regarding muscle power, results suggested moderating effects of the participants’ mean age with larger gains for older participants. In terms of flexibility, meta-regression results revealed moderating effects of the number of repetitions per exercise with higher numbers associated with larger gains and the time under stretching per session and total time under stretching with longer durations associated with larger benefits.

## Footnotes

1 The number of SS exercises per session was determined based on the assessment protocol used for muscle strength and power and whether the location and aim of the exercise was specific to the assessment protocol (e.g., for the assessment of the maximal voluntary contraction torque of the knee flexors, only exercises that stretched muscles of the lower extremities were considered).

2 Because of high correlations between the number of repetitions per session and the number of repetitions per week on one side (*r* = 0.742) and with the total number of repetitions on the other side (*r* = 0.826), we only considered the number of repetitions per session in the analysis.

3 Confidence intervals not overlapping 0 for the main effects or each other in the subgroup analysis are referred to as “clear” effects, while overlapping confidence intervals are referred to as “unclear” effects.

## Declarations

### Availability of data and material

The datasets generated during and/or analyzed in the current study as well as the supplementary materials are available in the Open Science Framework repository. All documents can be consulted at the following link: https://osf.io/gu9w6/.
